# One Pot Photomediated Formation of Electrically Conductive Hydrogels

**DOI:** 10.1021/acspolymersau.3c00031

**Published:** 2023-12-08

**Authors:** Dan My Nguyen, Chun-Yuan Lo, Tianzheng Guo, Taewook Choi, Shalini Sundar, Zachary Swain, Yuhang Wu, Charles Dhong, Laure V. Kayser

**Affiliations:** † Department of Chemistry and Biochemistry, University of Delaware, Newark, Delaware 19716, United States; ‡ Department of Materials Science and Engineering, University of Delaware, Newark, Delaware 19716, United States; § Department of Biomedical Engineering, University of Delaware, Newark, Delaware 19716, United States

**Keywords:** conductive hydrogels, photo-cross-linking, bioelectronics, PEDOT:PSS, oxidative polymerization, coumarin

## Abstract

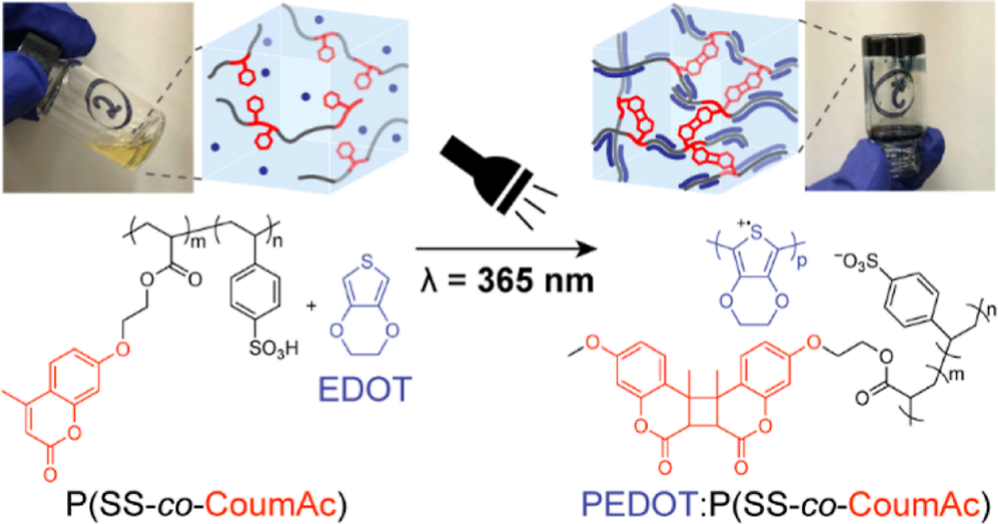

Electrically conductive hydrogels represent an innovative platform for the development of bioelectronic devices. While photolithography technologies have enabled the fabrication of complex architectures with high resolution, photoprinting conductive hydrogels is still a challenging task because the conductive polymer absorbs light which can outcompete photopolymerization of the insulating scaffold. In this study, we introduce an approach to synthesizing conductive hydrogels in one step. Our approach combines the simultaneous photo-cross-linking of a polymeric scaffold and the polymerization of 3,4-ethylene dioxythiophene (EDOT), without additional photocatalysts. This process involves the copolymerization of photo-cross-linkable coumarin-containing monomers with sodium styrenesulfonate to produce a water-soluble poly(styrenesulfonate-*co*-coumarin acrylate) (P(SS-*co*-CoumAc)) copolymer. Our findings reveal that optimizing the [SS]:[CoumAc] ratio at 100:5 results in hydrogels with the strain at break up to 16%. This mechanical resilience is coupled with an electronic conductivity of 9.2 S m^–1^ suitable for wearable electronics. Furthermore, the conductive hydrogels can be photopatterned to achieve micrometer-sized structures with high resolution. The photo-cross-linked hydrogels are used as electrodes to record stable and reliable surface electromyography (sEMG) signals. These novel photo-cross-linkable polymers combined with one-pot PEDOT (poly-EDOT) polymerization open possibilities for rapidly prototyping complex bioelectronic devices and creating custom-designed interfaces between electronics and biological systems.

## Introduction

Implantable^1−3^ and wearable^2,4−6^ bioelectronic devices enable seamless communication and interaction between biological systems and machines. Among these devices, bioelectronics based on electrically conductive hydrogels hold significant promise due to their unique material structures and properties. Electrically conductive hydrogels consist of cross-linked polymeric networks percolated with electrically conductive nanoparticles^7−11^ or polymers.^10−14^ Conductive hydrogels offer several advantages over traditional electronic materials. First, because of the cross-linked polymeric network, the elastic modulus of soft conductive hydrogels can be tuned to match those of biological tissues and the human body.^15,16^ Second, due to the presence of an electrically conductive phase and solvated ions in the porous network, conductive hydrogels can transduce ionic biological signals to electronic devices for sensing^17−19^ and vice versa for stimulation.^6,20,21^

Conductive hydrogels made from conducting polymers (CPs), such as poly(3,4-ethylenedioxythiophene) (PEDOT),^22,23^ polypyrrole (PPy),^24−26^ or polyaniline (PANI),^26,27^ offer superior compatibility with biological systems compared to conventional nanocomposite hydrogels like metal nanowires (NWs)^28,29^ and carbon nanotubes (CNTs).^30^ The enhanced compatibility arises from the conductive polymers’ inherent flexibility and their ability to facilitate both ionic and electronic conductivity, making them ideal candidates for advanced bioelectronic interfaces. However, bioelectronic devices made from conductive hydrogels are currently limited by their fabrication methods and our ability to control the shape and position of the hydrogel. Conventional methods such as molding^18,31,32^ have been frequently used to fabricate conductive hydrogels but can only provide simple structures with low resolution (>100 μm).^33^ This large size may not be compatible with bioelectronic applications requiring small electrodes. Alternatively, 3D-printing technologies, including nozzle-based printing^34−36^ and light-based printing,^36−38^ are capable of achieving more complex architectures with higher resolution. Nozzle-based printing uses nozzles to extrude conductive polymer ink into the desired architecture. The structure and resolution of conductive hydrogels greatly depend on the rheological properties of precursor inks.^33,34^ However, the choice of nozzle radius, polymer ink, and heating temperature can lead to reduced homogeneity and introduction of voids in the printed products, ultimately compromising the quality and structural integrity of the final objects.^36^

To achieve high-resolution spatial and temporal control, light-based printing methods of conductive hydrogels are emerging.^38−40^ This method of photogelation simultaneously patterns and cross-links conducting polymer solutions by light irradiation. However, photoprinting conductive hydrogels is still a challenging task because the conductive polymer absorbs UV light which may prevent photoactivation and cross-linking. To address this challenge, several studies have reported the fabrication of composite hydrogels of PEDOT:PSS and polyethylene glycol diacrylate (PEGDA) as a photocurable cross-linker using a visible-light photoinitiator system.^37,40−42^ The resulting hydrogels are patternable but usually show low electrical conductivity, likely due to low loadings of conductive polymer in an insulating scaffold. Instead, Wei et al. recently reported the preparation of 3D-printed conductive hydrogels by photopolymerization of the EDOT monomer.^38^ Their method is not direct photoprinting but a multistep process where the precursor ink was first extruded and then crosslinked by blue-light irradiation on nozzles under a phenol-coupling mechanism. Ruthenium [Ru] catalyzed both the photopolymerization and the phenol-coupling reaction. In addition to the need for a multistep and nozzle extrusion process, the use of [Ru] may also cause potential incompatibility with biological tissues due to its cytotoxicity.

**Figure 1:**
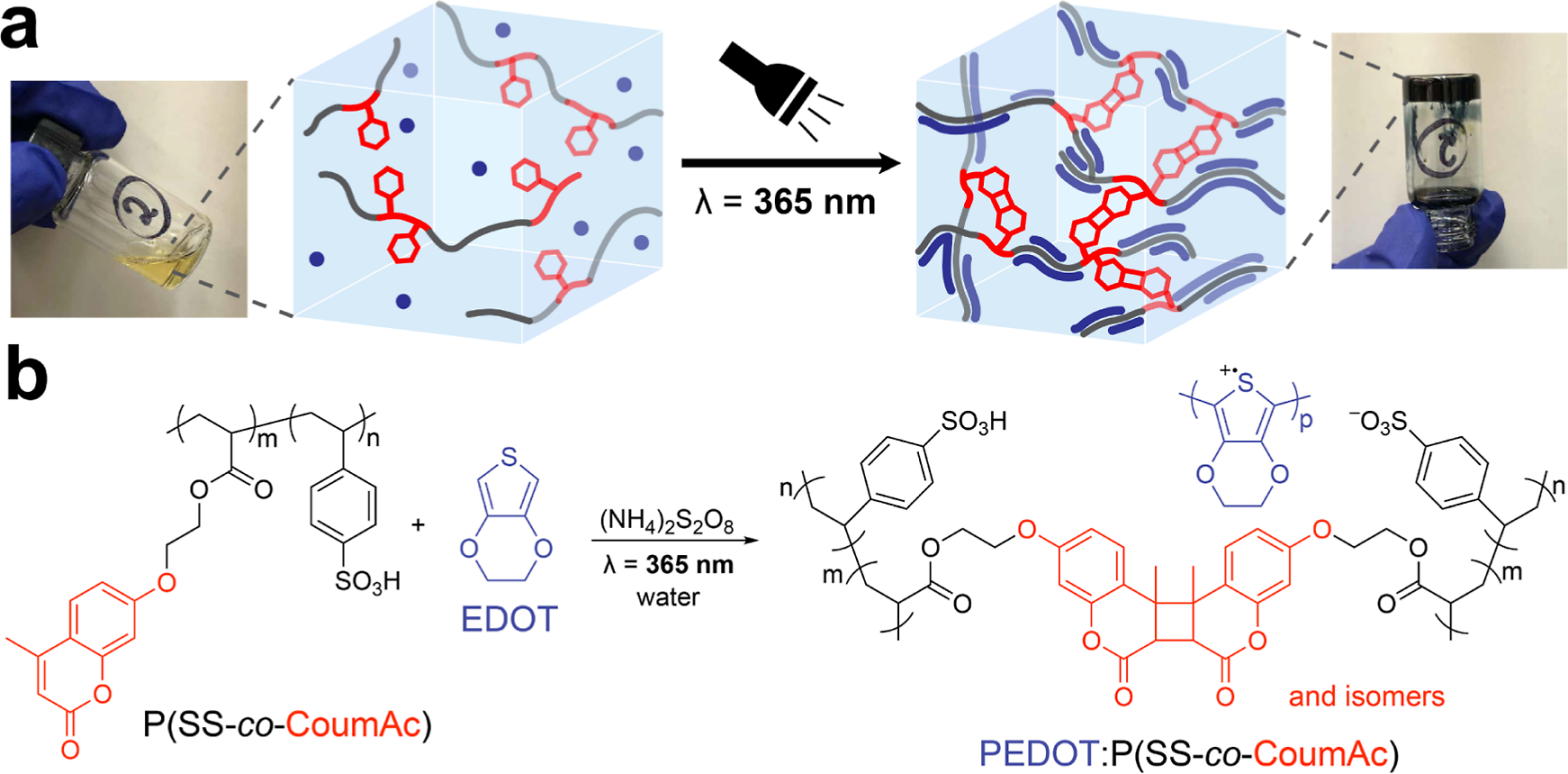
Overview of the photomediated formation of conductive hydrogels. (a) Photographs and schematics of the precursor solution (left) and the resulting conductive hydrogel after light irradiation (right). (b) Simultaneous photo-cross-linking of the coumarin-derived copolymer P(SS-*co*-CoumAc), which undergoes a [2 + 2] cycloaddition and an oxidative polymerization of EDOT.

Most of the photo-cross-linking methods explored to date to generate conductive hydrogels rely on the use of photoactive catalysts to generate radicals that will subsequently trigger cross-linking by radical addition or polymerization. The addition of photocatalysts, however, often complicates the process as it requires the removal of oxygen and either needs to be biocompatible or removed after the hydrogel formation. Alternatively, hydrogels based on covalent chemistry that undergoes dimerization processes under light irradiation but without a chemical catalyst such as coumarins^43−45^ could address this problem. Under long-wave UV irradiation (365 nm), coumarins undergo a [2 + 2] cycloaddition. Therefore, attaching coumarin groups on a polymer backbone enables cross-linked hydrogels through the formation of coumarin dimers. While the use of coumarins has been reported before for nonconductive hydrogels,^43−48^ translation to systems with conductive polymers has not been shown, likely because the conducting polymers typically absorb light at around the same wavelength, thereby preventing cross-linking. To address this problem, we report herein the simultaneous photo-cross-linking of a polymeric scaffold and the polymerization of a conducting polymer under light irradiation. Our approach involves the copolymerization of coumarin-containing monomers with sodium styrenesulfonate (NaSS) to yield water-soluble poly(styrenesulfonate-*co*-coumarin acrylate) (P(SS-*co*-CoumAc)). Under UV irradiation (365 nm), the transparent, nonconducting solution of P(SS-*co*-CoumAc), 3,4-ethylene dioxythiophene (EDOT), and ammonium persulfate (APS) transforms into a dark-blue, electronically conducting hydrogel (Figure 1a). The formation of the conductive hydrogel relies on the simultaneous covalent photo-cross-linking of PSS copolymers with coumarin-derived monomers and the oxidative polymerization of EDOT (Figure 1b). In this system, we found that the polymerization of EDOT did not require the addition of a [Ru] photocatalyst. The resulting conductive hydrogels exhibit both ionic and electronic conductivity, possess tunable elastic moduli in the MPa range, and display a reasonable strain at break (up to 16%). We showed that the conductive hydrogels can be photoprinted using a conventional photolithography system. In future work, we expect that this photomediated formation of conducting hydrogels applied to stereolithography could enable the printing of 3D networks with a controlled microstructure and provide materials for new applications in bioelectronics.

## Experimental Methods

### Synthesis of 7-(2-Hydroxyethoxy)-4-methylcoumarin (CoumOH)

CoumOH was synthesized according to a previously reported procedure.^44^ 4-Methylumbelliferone (4.00 g, 22.7 mmol, 1 equiv) and potassium carbonate (6.23 g, 45.4 mmol, 2 equiv) were mixed in anhydrous DMF (40 mL) under a nitrogen atmosphere. 2-Bromoethanol (2.42 mL, 34.0 mmol, 1.5 equiv) was added, and the reaction mixture was heated at 90 °C for 18 h. It was then cooled to room temperature and poured into ice-cold water. The white precipitate was collected by vacuum filtration and dried in vacuo. Yield: 4.50 g (90%). ^1^H NMR (600 MHz, DMSO-*d*_6_): δ (ppm) 7.70–7.68 (dd, 1H), 6.99–6.97 (m, 2H), 6.21 (d, 1H), 4.92 (t, 1H), 4.10 (t, 2H), 3.75 (m, 2H), 2.40 (d, 3H) (Figure S1).

### Synthesis of 7-(2-Acryloyloxyethoxy)-4-methylcoumarin (CoumAc)

CoumAc was synthesized according to a previously reported procedure.^44^ 7-(2-Hydroxyethoxy)-4-methylcoumarin (4.00 g, 18.2 mmol, 1 equiv) was suspended in chloroform (75 mL) and triethylamine (5.06 mL, 36.3 mmol, 2 equiv) was added. Acryloyl chloride (2.94 mL, 36.3 mmol, 2 equiv) was added portionwise under argon, and the reaction mixture was stirred at room temperature. After 1 h, additional triethylamine (2.53 mL, 18.2 mmol, 1 equiv) and acryloyl chloride (1.47 mL, 18.2 mmol, 1 equiv) were added and the solution was stirred overnight. The reaction mixture was diluted with dichloromethane, washed with brine (100 mL ×2), dried over sodium sulfate, filtered, and concentrated in vacuo. The crude product was recrystallized twice from ethanol to yield CoumAc as a white powder. Yield: 1.83 g (37%). ^1^H NMR (600 MHz, CDCl_3_): δ (ppm) 7.45 (d, 1H), 6.82 (dd, 1H), 6.77 (d, 1H), 6.39 (dd, 1H), 6.11 (m, 2H), 5.81 (dd, 1H), 4.48 (t, 2H), 4.21 (t, 2H), 2.34 (s, 3H) (Figure S2).

### Synthesis of P(SS-*co*-CoumAc) Copolymer

CoumAc (307.5 mg, 1.12 mmol) and NaSS taken in different amounts according to the desired ratio of PSS to PCoumAc (1.04 g–5 mmol, 2.08 g–10.1 mmol, and 4.16 g–20.2 mmol for ratios of 100:5, 100:10, and 100:20, respectively) along with 4,4′-azobis(4-cyanovaleric acid) (ACVA) (147 mg, 0.52 mmol) were dissolved in a mixture of 16 mL of water and 8 mL of dioxane and degassed under a flow of nitrogen for 30 min. The reaction mixture was placed in an oil bath at 70 °C for 18 h. The reactions were stopped by rapid cooling and exposure to air. P(SSNa-*co*-CoumAc) was purified by dialysis against water for 2 days with multiple water bath changes. The purified solution was dried under vacuum to yield a solid product. Next, P(NaSS-*co*-CoumAc) was dissolved in water (0.1 g mL^–1^) and stirred over an acidic resin (Dowex Marathon C hydrogen form) for 6 h at room temperature to afford the acid form of P(SS-*co*-CoumAc). Then, P(SS-*co*-CoumAc) was filtered through a 0.45 μm nylon syringe filter and dried under vacuum. ^1^H NMR spectra of P(SS-*co*-CoumAc) are provided in Figures S3–S5, and SEC traces are provided in Figure S6.

### Preparation of PEDOT:P(SS-*co*-CoumAc) Conductive Hydrogels

P(SS-*co*-CoumAc) (150 mg, 50 wt %) was dissolved in 0.3 mL of water. Depending on the ratio of PSS to PCoumAc, different amounts of EDOT and APS were added to the solution to keep the optimized mole ratio of APS to EDOT of 1.2:1^49^ and the mole ratio of PSS to EDOT of 100:13. For the ratio of PSS to PCoumAc of 100:20, EDOT (8.25 μL, 0.078 mmol) and APS (21.3 mg, 0.093 mmol) were added. For the ratio of PSS to PcoumAc of 100:10, EDOT (9 μL, 0.085 mmol) and APS (22.5 mg, 0.099 mmol) were added. For the ratio of PSS to PcoumAc of 100:5, EDOT (9.45 μL, 0.0089) and APS (24.4 mg, 0.107 mmol) were added. The solutions were stirred vigorously at room temperature for 5 min and then irradiated with 365 nm UV light (25 mW cm^–2^) for 120 min in a sealed container or chamber until gelation was complete. The samples were washed by soaking in deionized (DI) water (3× 15–20 min) and phosphate-buffered saline (PBS) at pH 7.4 (3× 15–20 min) and then stored in the PBS solution overnight before electronic and mechanical measurements.

## Results and Discussion

Our goal in this study was to achieve photogelation of PEDOT:PSS-based conductive hydrogels. As such, we first synthesized a PSS copolymer with a previously reported photo-cross-linkable monomer, CoumAc. The P(NaSS-*co*-CoumAc) copolymers were synthesized by free-radical polymerization in a mixture of water and 1,4-dioxane at 70 °C with three different ratios of NaSS to CoumAc (100:20, 100:10, and 100:5, Table 1) to determine the impact of the cross-linker density on the mechanical properties of the resulting hydrogels and the gelation time. As determined by proton nuclear magnetic resonance (^1^H NMR), the incorporation of CoumAc in the final polymer was a little lower than the [SSNa]:[CoumAc] feed ratio, likely due to the slightly lower reactivity of the acrylate CoumAc monomer compared with the styrenic NaSS. It is interesting to note that we were able to achieve CoumAc loadings as high as 20 mol % without macroscopic gelation, a problem that was previously observed in copolymers of *N,N*-dimethylacrylamide with over 5 mol % CoumAc.^44^ The molecular weight and size distribution of copolymers, as measured by size exclusion chromatography (SEC), ranged from 24.5 kg mol^–1^ for the 100:20 copolymer to 37.8 kg mol^–1^ at lower CoumAc loadings. The higher viscosity of the reaction mixture at higher loadings of CoumAc likely explains the slightly lower molecular weights obtained. Given that the molecular weight of the three copolymers studied was roughly within the same range, we expect it to have only a small impact on the rheological and mechanical properties of hydrogels. After the formation of the P(NaSS-*co*-CoumAc) copolymer and in preparation for the incorporation of PEDOT, it was stirred over an acidic resin to replace sodium ions with protons, resulting in P(SS-*co*-CoumAc).

**Table 1:** Synthesis of P(NaSS-*co*-CoumAc) Copolymers

polymer name indicating SS:CoumAc molar ratio	[SSNa]:[CoumAc] feed^a^	[SS]:[CoumAc] actual^b^	*M*_*n*_^c^ (kg mol^–^^1^)	*D̵*^c^
100:20	100:22.2	100:18	24.5	1.92
100:10	100:11.1	100:10	30.2	2.33
100:5	100:5.6	100:4.3	37.8	3.44

a The initial molar feed ratio of SSNa to CoumAc as measured by ^1^H NMR spectroscopy.

b Ratio of SSNa to CoumAc as measured by ^1^H NMR spectroscopy of the purified copolymer.

c Measured by SEC in a mixture of a water buffer and DMF calibrated against PSSNa standards using a refractive index detector.

**Figure 2:**
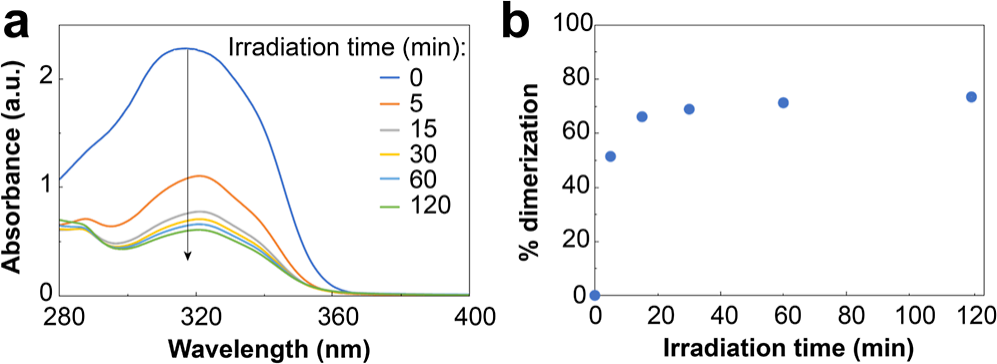
UV–vis study of the cross-linking of P(SS-*co*-CoumAc). (a) UV absorption spectra of a solution of P(SS-*co*-CoumAc) in water (0.05 wt %) irradiated at 365 nm over 120 min. (b) Calculated percentage of coumarin dimerization as a function of irradiation time at 365 nm.

Given that P(SS-*co*-CoumAc) had not been reported previously, we first needed to establish whether photogelation by [2 + 2] cycloaddition and cross-linking was feasible. In its monomeric form, coumarin exhibits a strong absorbance at 320 nm. When exposed to UV light (365 nm), coumarin undergoes a [2 + 2] cycloaddition reaction, resulting in the formation of a cyclobutane ring (Figure 1b) which is not UV-active. This dimerization typically leads to a decrease in the UV–vis absorption peak at 320 nm during the reaction.^43−45^ The photo-cross-linking of coumarin in P(SS-*co*-CoumAc) was therefore initially investigated by UV–vis spectroscopy. A diluted solution of P(SS-*co*-CoumAc) with a ratio of 100:10 (0.05 wt % in water) was prepared and exposed to 365 nm light (25 mW cm^–2^). Figure 2a shows the change in the UV–vis absorption over the course of irradiation. As expected, the absorbance at 320 nm diminished as the coumarin groups were dimerized. After 1 h, approximately 75% of the coumarin moieties had dimerized and reached their maximum (Figure 2b). However, due to the low concentration needed as to not saturate the UV–vis, these solutions did not form a gel.

The cyclobutane formed through dimerization can generally be cleaved back to the monomeric coumarin by irradiation of the solution at a wavelength of 254 nm. For P(SS-*co*-CoumAc), this reversible process was also feasible as seen by an increase in the 320 nm peak after irradiation at 254 nm for 5 min (Figure S7a). However, the absorption intensity did not fully return to its original level. After 15 min of irradiation, approximately 5% of the dimer still remained (Figure S7b). This phenomenon can be explained by the formation of an equilibrium between the open and closed forms of coumarin at this wavelength.^50^

**Figure 3:**
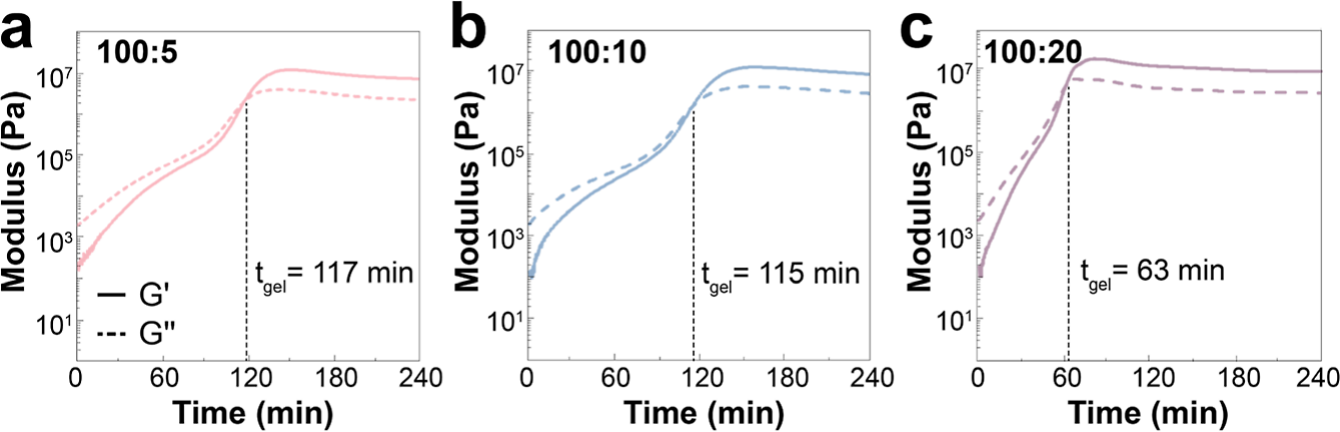
Photorheology measurements at 365 nm as a function of irradiation time of a solution of P(SS-*co*-CoumAc) in water (50 wt %) with SS:CoumAc cross-linker ratios of (a) 100:5, (b) 100:10, and (c) 100:20.

Based on these results, the polymer concentration was increased to investigate the gelation behavior. At 40 wt %, the solution of P(SS-*co*-CoumAc) became a soft gel when exposed to 365 nm for 120 min. We therefore decided to increase the concentration to 50 wt % for the next experiments. At that concentration, we observed the formation of a stable gel within 120 min (Figure S8). To achieve a more quantitative assessment of the gelation time, we monitored real-time changes in storage modulus (*G*′) and loss modulus (*G*″) by photorheology under irradiation at 365 nm (Figure 3). In all three copolymers, the initial state was liquid, with *G*′ being lower than *G*″. However, upon light exposure, both *G*′ and *G*″ increased, until *G*′ surpassed *G*″, indicating the transformation from the liquid precursor to a hydrogel state. Consequently, the time at which *G*′ equaled *G*″ was defined as the gelation time (*t*_gel_) of the hydrogels. The gelation times for the pristine hydrogels with ratios 100:5, 100:10, and 100:20 were 117, 115, and 63 min, respectively. As the amount of coumarin cross-linker increased, the gelation time decreased, leading to a faster conversion of the precursor liquid to a hydrogel state. These gelation times were slower than the kinetics of the cycloaddition obtained from the UV–vis experiments (the reaction reached equilibrium within 20–30 min). This observation might be explained by the high viscosity of the precursor solutions (400 Pa·s) at the concentration needed for gelation in the rheology experiments. Surprisingly, the storage modulus remained similar in all three cases at around 7 MPa, which suggests a similar degree of cross-linking in all three formulations Again, we believe that the cross-linking is limited by the high viscosity of the reaction, likely leading to a similar maximum number of coumarin dimer cross-links.

Having confirmed that the precursor copolymer can be photo-cross-linked, we then studied the incorporation of the conducting polymer PEDOT^+^. PEDOT was chosen for its high conductivity and stability in water and oxygen. To achieve the one-pot photomediated preparation of conductive hydrogels, we hypothesized that the long-wave UV light (365 nm) could simultaneously enable the photo-cross-linking of the coumarin-derived copolymer P(SS-*co*-CoumAc) and drive the photocatalyzed oxidative polymerization of EDOT. Previous studies have shown that EDOT monomers and other aromatic heterocycles, such as pyrrole and thiophene derivatives, can be oxidatively polymerized in the presence of a photoredox catalyst such as [Ru(bpy)_3_]^2+^.^38,51−55^ Upon long-wave light irradiation, [Ru(bpy)_3_]^2+^ reaches a photoexcited state *[Ru(bpy)_3_]^2+^ which, in the presence of electron acceptors, generates a strong oxidant, [Ru(bpy)_3_]^3+^, capable of triggering the oxidative polymerization of aromatic heterocycles. As mentioned in the Introduction, Wei et al. used this redox chemistry to achieve the photocatalyzed polymerization of EDOT in 3D extruded inks.^38^ To start our studies for the photomediated formation of PEDOT:PSS-*co*-CoumAc gels, we therefore used similar reaction conditions for the photopolymerization of EDOT in P(SS-*co*-CoumAc) (100:10 ratio chosen for its intermediate loading of CoumAc) (50 wt % in water) with 0.33 mM of [Ru(bpy)_3_]^2+^, 93 mM of EDOT (SS:EDOT ratio of 100:4), 110 mM of APS, and light irradiation at 365 nm (25 mW cm^–2^). We initially monitored visually for the gelation of the solution, and the polymerization of PEDOT^+^ was seen by the characteristic color change from yellow (monomer) to green (oligomers) to dark blue (doped PEDOT) (Table 2 and Figure S9). Under these conditions, we observed the formation of a dark blue gel in 90 min (Figure S9a and Table 2, entry 1) with the solution turning dark blue in less than 15 min, proving that the one-pot, photomediated formation of PEDOT hydrogels was possible. The gelation time of 90 min was consistent with that necessary for coumarin photodimerization but slightly faster than without EDOT present (Figure 3b). This observation may be explained by a synergistic effect from the coumarin dimerization and PEDOT polymerization within the covalently cross-linked network to form gels faster, likely in the form of an interpenetrating network. In control experiments, we then investigated the impact of the presence of coumarin cross-linker, UV light, APS, and the Ru(II) catalyst on the formation of PEDOT^+^ hydrogels (Table 2, entry 2). In the absence of coumarin units (only PSS, Figure S9b), the solutions turned dark blue but did not gel within a 90 min time frame despite the apparent increase in the viscosity of the solution. This observation is consistent with the polymerization of PEDOT but in the absence of intermolecular coumarin cross-links. In the absence of light (Figure S9c and Table 2, entry 3), no gelation was observed, and the PEDOT polymerization proceeded very slowly with a faint green coloration only seen after 30 min. This experiment indicated that light is crucial for both the cross-linking and the polymerization. Without the APS oxidant (Figure S9d, and Table 2, entry 4), polymerization still proceeded but more slowly and likely only formed oligomers. No gelation was observed within 90 min, which is consistent with the rheology experiments without EDOT, which showed that gelation only occurred after 120 min if polymeric PEDOT is not present to accelerate gelation. The control experiment in the absence of added Ru(II) catalyst gave the most surprising result (Figures 1a and S9e and Table 2, entry 5). PEDOT polymerization proceeded roughly at the same rate, and gelation was observed within the same time frame as with the Ru(II) photoredox catalyst. Three possibilities might explain this observation. The reaction could be accelerated by heat generated from the lamp, and there could be traces of catalytic oxidant in the reaction mixture, or oligomeric PEDOT—formed by early stage oxidation with APS—that could act as a photocatalyst^56−58^ and lead to further polymerization via a self-catalyzed reaction pathway. To investigate if temperature was responsible for this acceleration in the polymerization, we monitored the temperature of the reaction mixture under the UV lamp and found it to be around 28–30 °C. We also performed the reaction in the absence of light but with heating at 50 °C (Figure S9f, and Table 2, entry 6). As expected, no gel was formed after 90 min. The polymerization of PEDOT was slower than with light irradiation (Figure S9e) but faster than the reaction performed at room temperature without light (Figure S9c). With these control experiments, we cannot completely rule out nor validate that heat was responsible for the faster PEDOT polymerization kinetics, nor can we confirm that the polymerization is purely photocatalyzed. At this time, the presence of trace metals that could catalyze the PEDOT photopolymerization remains a possibility, and additional studies, outside the scope of this paper, will need to be performed to confirm the reaction mechanism. But, the result remains that additional photoredox catalysts are not needed for the photomediated formation of conductive hydrogels. Given the potential cytotoxicity of ruthenium and the difficulty in removing traces of metals in hydrogels, we decided to continue this study without [Ru(bpy)_3_]^2+^.

**Table 2:** Control Experiments for the Photomediated Formation of Conductive Hydrogels

entry	supporting polymer	[Ru] (mM)	UV (365 nm)^a^	APS (mM)	EDOT polymerization at 15 min	EDOT polymerization at 90 min	gelation at 90 min
1	P(SS-co-CoumAc)	0.33	yes	110	yes	yes	yes
2	PSS	0.33	yes	110	yes	yes	no
3	P(SS-co-CoumAc)	0.33	no	110	no	yes	no
4	P(SS-co-CoumAc)	0.33	yes	0	starting	yes	no
5	P(SS-co-CoumAc)	0	yes	110	yes	yes	yes
6	P(SS-co-CoumAc)	0.33	no (50°C)	110	starting	yes	no

a The reactions were performed without additional external heating unless otherwise noted (entry 6).

**Figure 4:**
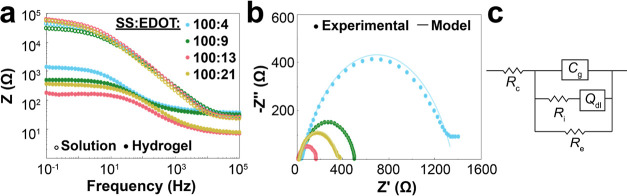
EIS as a function of the loading of EDOT in P(SS-*co*-CoumAc) at a 100:10 ratio. (a) Bode plots of precursor solutions and conductive hydrogels after 120 min of light irradiation and washed with both DI water and PBS solution. (b) Nyquist plots of the conductive hydrogels. (c) Equivalent circuit model used to fit the experimental data.

With general reaction conditions in hand to obtain photo-cross-linked hydrogels, the effect of the loading of EDOT on the electronic properties of the conductive hydrogels was investigated by electrochemical impedance spectroscopy (EIS) and 2-point parallel plate resistance measurements. From a molar ratio of SS:EDOT of 100:4 in the initial studies, the loading of EDOT was increased to 100:9, 100:13, and 100:21. The solutions were irradiated for 120 min to ensure complete gelation and EDOT polymerization, determined based on when the moduli in photorheology experiments reached a plateau, and then washed with deionized water and PBS solution. Bode plots from EIS (Figure 4a) showed that, compared to precursor solutions, all the conductive hydrogels displayed significantly lower impedance (over 2 orders of magnitude), especially in the low-frequency range. This decrease in impedance is consistent with the incorporation of interconnected conducting polymer chains (PEDOT^+^) into the resulting hydrogels. Notably, at an identical gel volume, the hydrogel with a 100:13 ratio of SS to EDOT displayed the lowest impedance magnitude. The Nyquist plot of the hydrogels (Figure 4b) were analyzed and fitted to the equivalent circuit model commonly used for PEDOT-based conductive hydrogels (Figure 4c).^31,32,34^ The tabulated values extracted from this model are presented in Table 3 and show small χ^2^ values, indicating a good fit for the model. The values of *R*_c_, *R*_e_, and *R*_i_ are, respectively, the resistive contributions from the assembled cell used for the test and the electronic and ionic resistance from the conductive hydrogel. *C*_g_ is the ideal geometric capacitance of conductive hydrogels and *Q*_dl_ is the constant phase element describing the nonideal double-layer capacitance. The values reported in Table 3 represent the average values for *R*_e_ and *R*_i_ from three independently prepared samples, with *R*_e_ being the lowest for the 100:13 ratio (160 Ω) and *R*_i_ being the lowest for the 100:21 ratio (5 Ω). Additionally, the conductivity, measured using the 2-point parallel plate method, showed the highest value for the 100:13 ratio (4.58 ± 0.12 S m^–1^), comparable to other PEDOT-based hydrogels.^32,34,38,59^

**Table 3:** Electronic Properties of the Conductive Hydrogels

SS:EDOT molar ratio	[EDOT] (mM)	measured PSS:PEDOT ratio^a^	conductivity^b^ (S m^–^^1^)	*R*_e_^c^ (Ω)	*R*_i_^c^ (Ω)	χ^2^
100:4	93	100:4.2	0.57 ± 0.006	1364	17	0.03
100:9	186	100:6.9	1.69 ± 0.03	473	14	0.01
100:13	279	100:8.3	4.58 ± 0.12	160	11	0.05
100:21	465	100:10.5	1.15 ± 0.06	341	5	0.04

a Obtained by XPS.

b Obtained by two-point parallel plate measurement.

c Obtained by fitting EIS data against the equivalent circuit model in Figure 4c.

In addition to the color change, the presence of PEDOT^+^ in the bulk of the resulting hydrogels was also confirmed by X-ray photoelectron spectroscopy (XPS) of freeze-dried samples (Figure S10). The S (2p) electrons of PEDOT and PSS have different binding energies, so the ratio of PSS:PEDOT can be determined by XPS.^60^ The S (2p) peaks at a binding energy of 169 eV correspond to the sulfur in the sulfonate groups of PSS, and the S (2p) signals at 165 eV correspond to the sulfur in the thiophene fragment of PEDOT. The area ratio of the S (2p) peaks can therefore be used to estimate the relative composition of PSS to PEDOT of samples. The ratios are just slightly lower than the ratio of SS to EDOT added (Table 3), indicating that some fraction of EDOT/PEDOT is washed away during the hydrogel workup steps. We also observed the presence of both PEDOT^0^ and PEDOT^+^ by XPS, which show S (2p) peaks at 163 and 166–167 eV, respectively.^61^ While calculating the degree of oxidation of PEDOT in the samples is theoretically possible, the low fraction of PEDOT in the gels prevented us from doing an accurate peak deconvolution. Based on the EIS and conductivity measurements, we selected the optimized SS:EDOT ratio of 100:13 for the preparation of subsequent conductive hydrogels.

**Figure 5:**
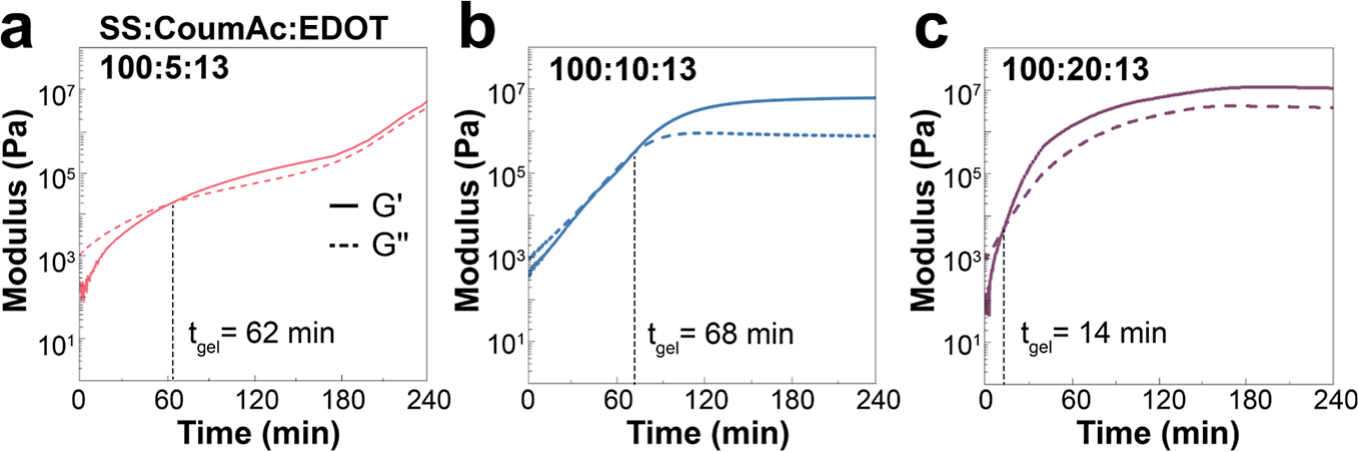
Photorheology measurements at 365 nm as a function of irradiation time of a solution of P(SS-*co*-CoumAc) (50 wt %), EDOT, and APS in water with SS:CoumAc:EDOT ratios of (a) 100:5:13, (b) 100:10:13, and (c) 100:20:13.

The solgel transition (Figure 5) and change in viscosity (Figure S11) during photoirradiation were then monitored using photorheology measurements at a constant SS:EDOT ratio of 100:13 but with a varying loading of CoumAc in P(SS-*co*-CoumAc). Compared to the hydrogels without PEDOT, the gelation process and the increase in viscosity of the conductive hydrogels were significantly accelerated (approximately 50 min faster). This result was consistent with our previous experiments, showing that gelation was not only due to coumarin cross-linking but also due to the formation of interconnected PEDOT chains. The gelation times for the conductive hydrogels with SS:CoumAc ratios of 100:5, 100:10, and 100:20 were 62, 68, and 14 min, respectively. This acceleration in the gelation, compared with the experiments in Figure 3, can be explained by the formation of the PEDOT polymer within the network. PEDOT is not soluble in water, and it is common that in the absence of a sufficient amount of a polyelectrolyte counterion and/or stirring to form a colloidal suspension, PEDOT either precipitates or gels when polymerized in water. In this case, we believe that PEDOT formed within the hydrogel network, which accelerated the gel formation through the formation of a water-insoluble polymer. As the amount of coumarin cross-linker increased, the gelation time decreased, resulting in a more rapid conversion of the precursor liquid into a hydrogel state. Notably, the conductive hydrogel with a ratio of SS:CoumAc:EDOT of 100:20:13 achieved gelation within a time frame which could be suitable for stereolithography (14 min for 0.5 mL) (Figure 5c). At low loadings of CoumAc (Figure 5a), the moduli did not reach a plateau within the 240 min experiment. We posit that the formation of PEDOT prevented some dimerization of CoumAc. As PEDOT formed, the solutions became very dark, with PEDOT likely absorbing the light that would have been necessary for the coumarin cross-linking to happen. But both 100:10:13 (Figure 5b) and 100:20:13 (Figure 5c) reached equilibrium at roughly 120 min, indicating that both the coumarin cross-linking and PEDOT polymerization were completed within this time. The final moduli of these two conductive hydrogels were close to that of the parent hydrogel without EDOT (*G*′ ∼ 10^7^ Pa, Figure 3b,c) indicating that at those higher CoumAc loadings, cross-linking was sufficiently fast to outcompete the formation of light-absorbing PEDOT. We should note that when we tried to decrosslink the conductive hydrogels by irradiating at 254 nm, we could not see any evidence for degelation. Again, it is likely because PEDOT absorbed light too strongly and prevented the coumarin retro-cycloaddition.

**Figure 6:**
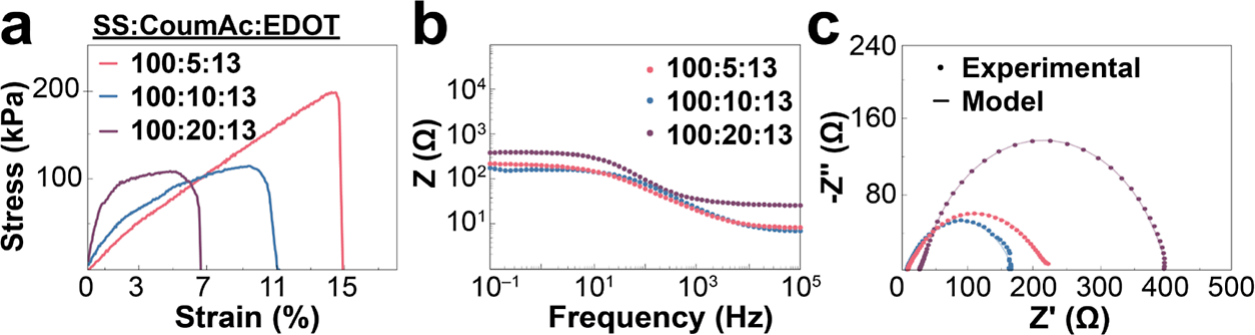
Effect of the proportion of CoumAc cross-linker on the mechanical and electronic properties of the PEDOT:P(SS-*co*-CoumAc) conductive hydrogels. (a) Representative tensile test. (b) Bode plots, and (c) Nyquist plots from EIS studies.

The influence of the proportion of coumarin cross-linker on the mechanical properties of PEDOT:P(SS-*co*-CoumAc) conductive hydrogels was examined through tensile tests. Still keeping a SS:EDOT ratio of 100:13, the solutions were irradiated for 120 min in a mold, and the hydrogels were washed with DI water and PBS prior to the tensile test. Figure 6a illustrates the stress–strain curves obtained from the tensile elongation of hydrogels with different SS:CoumAc ratios. The hydrogels exhibited varying elastic moduli: 4.6 5.4, and 10.5 MPa for ratios of 100:5, 100:10, and 100:20, respectively (Table 4). As the amount of cross-linker increased, the elastic modulus of the hydrogels increased, while the toughness and strain at break decreased. Among the ratios tested, the hydrogel with a ratio of 100:5 displayed the highest strain at break (16%). However, at 120 min irradiation, it is likely that the hydrogel network was not fully cross-linked, which may explain the relatively lower elastic modulus compared with the other two formulations. Lastly, we determined the stability of the conductive hydrogel in 1× PBS by measuring the amount of swelling and stability under ambient conditions by measuring deswelling/drying in air. Figure S12 shows that the weight percentage of the conductive hydrogel, when submerged in 1× PBS, increased gradually over 6 h to reach 110 wt %. The gels remained at this mass for 24 h. When left under ambient conditions, the weight percentage of the conductive hydrogel decreased and reached a plateau at 60 wt % after 10 h, consistent with water evaporation. The conductive hydrogel remained at 60 wt % after a week.

**Table 4:** Tabulated Mechanical and Electronic Properties of PEDOT:P(SS-*co*-CoumAc) Conductive Hydrogels Showing the Effect of the Amount of CoumAc Crosslinker

SS:CoumAc ratio	elastic modulus (MPa)^a^	strain at break(%)^a^	toughness (kJ m^–^^3^)^a^	conductivity (S m^–^^1^)^b^	*R*_e_ (Ω)^c^	*R*_i_ (Ω)^c^	χ^2^
100:20	7.2 ± 3.0	6 ± 2	6.0 ± 2.4	2.8 ± 0.1	293 ± 61	4.7 ± 0.6	0.008
100:10	5.4 ± 3.6	13 ± 5	9.0 ± 3.2	4.6 ± 0.1	166 ± 6	9.2 ± 1.7	0.006
100:5	4.6 ± 2.0	16 ± 1	16.2 ± 3.0	9.2 ± 0.7	198 ± 24	9.0 ± 2.5	0.03

a Measured by tensile tests.

b Obtained by two-point parallel plate measurements.

c Obtained by EIS by fitting to the equivalent circuit model shown in Figure 4c. Average of three separate samples.

To gain further insights into the effect of the proportion of coumarin cross-linker on the ionic and electronic conductivities, EIS and parallel plate resistance measurements were conducted. Bode plots (Figure 6b) showed that the impedance magnitude was just slightly lower in the formulations with a less amount of coumarin cross-linker. Nyquist plots (Figure 6c) of the hydrogels were analyzed and fitted to the equivalent circuit model shown in Figure 4c. For all three ratios tested, *R*_e_ and *R*_i_ were roughly the same with *R*_e_ around 200 Ω and *R*_i_ of 5–9 Ω. The lack of major differences between the three formulations was not surprising because the ratio of PEDOT:PSS was kept constant at 13:100, and PEDOT^+^ was the only contributor to electronic conductivity in the hydrogels. The electronic conductivities, measured through 2-point parallel plate measurements, showed only a slightly higher value (9.2 S m^–1^) in the hydrogels with the lowest amount of cross-linker (SS:CoumAc 100:5).

**Figure 7:**
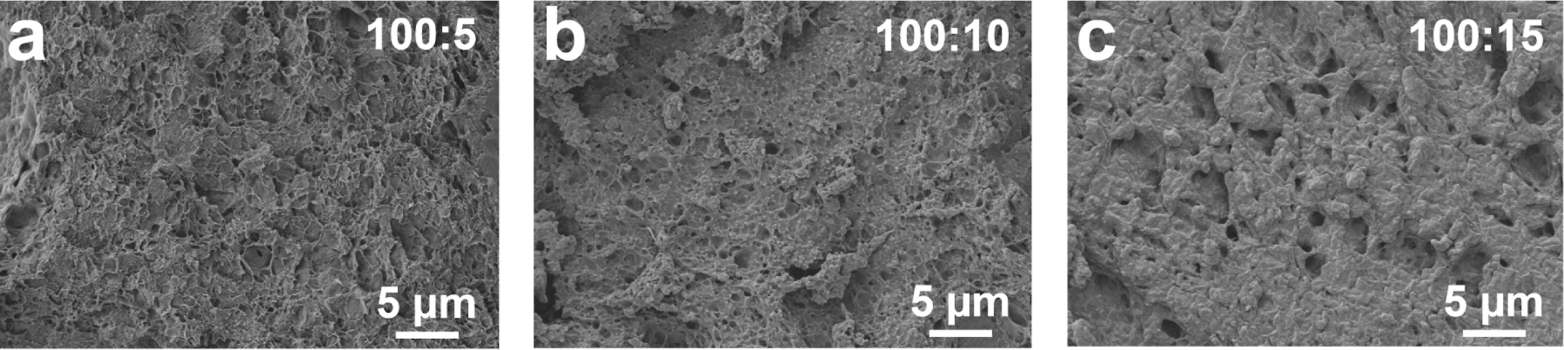
SEM images of cross sections of freeze-dried conductive hydrogels with SS:CoumAc ratios of (a) 100:5, (b) 100:10, and (c) 100:20.

Next, the microstructure of the hydrogels was studied by scanning electron microscopy (SEM) on freeze-dried cross sections. The SEM images of PEDOT:P(SS-*co*-CoumAc) hydrogels with SS:CoumAc cross-linker ratios of 100:5 (Figure 7a), 100:10 (Figure 7b), and 100:20 (Figure 7c) revealed a 3D interconnected porous structure. The cross-sectional SEM image of all hydrogels showed a dense but relatively homogeneous network. The average pore size, as determined by image analysis, of the conductive hydrogels of ratios 100:5, 100:10, and 100:20 were 0.77 ± 0.19 μm, 0.51 ± 0.14 μm, and 0.47 ± 0.22 μm, respectively. These values are consistent with an increase in the network density at higher degrees of cross-linking. As the degree of coumarin cross-linker increased, we also saw a change from a fibrillar network morphology to the formation of denser aggregates, possibly due to PEDOT aggregation. This change in morphology could explain the difference in the mechanical behavior under tensile tests (Figure 6a), where the fibrillar networks are more elastic (100:5 ratio), and the aggregated networks are getting more plastically deformed (100:20).

**Figure 8:**
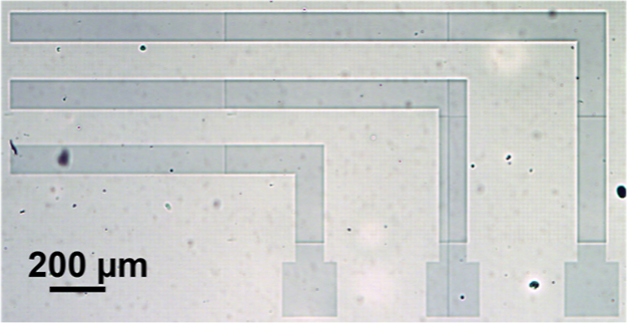
Optical microscopy image of a photoprinted electrode-like pattern with a 100 μm width. Thickness was roughly 500–800 μm.

As a proof of concept for the utility of photomediated conductive hydrogel formation, we used a commercial maskless photolithography tool (Smart Print UV, Microlight 3D) to test if our formulation can achieve photopatterned conductive hydrogels. For this experiment, we chose the formulation with the fastest gelation time (Figure 5c), SS:CoumAc:EDOT 100:20:13. Once EDOT and APS were mixed in the P(SS-*co*-CoumAc) aqueous solution, the formulation was stable in the dark at room temperature for about 30 min before EDOT started reacting, as seen by the appearance of a green color. The precursor solution was drop-cast and spread manually on a glass slide pretreated with plasma to increase its hydrophilicity. The glass slide was then placed on the exposure stage under the UV light source which used a wavelength of 385 nm at an intensity of 2072 mW cm^–2^ to create a design with features that had a minimum width of 100 μm through 45 s exposure times. The total printing time to achieve the desired pattern was 13.5 min. As seen in the microscopy image (Figure 8), blue patterns with a good resolution were obtained. No “bleeding” of PEDOT was observed outside the patterned features, which suggests that its diffusion was limited by the formation of a cross-linked network even at these short exposure times. The films sufficiently well adhered to the glass slide, thanks to the plasma treatment. However, to ensure a higher mechanical stability and adhesion, we would recommend using a glass surface treatment with an adhesive monolayer such as (3-glycidyloxypropyl)trimethoxysilane (GOPS).^62^ While this photoprinting experiment was not fully optimized, it showed that we can achieve patterns of conductive hydrogels with micron-scale resolution. Higher resolution (down to submicrons) could be obtained by two-photon stereolithography.^63^ We expect that stereolithography using a digital mirror device (DMD)^64^ and this ink formulation would enable the 3D printing of conductive hydrogels, which will be explored in future publications.

**Figure 9:**
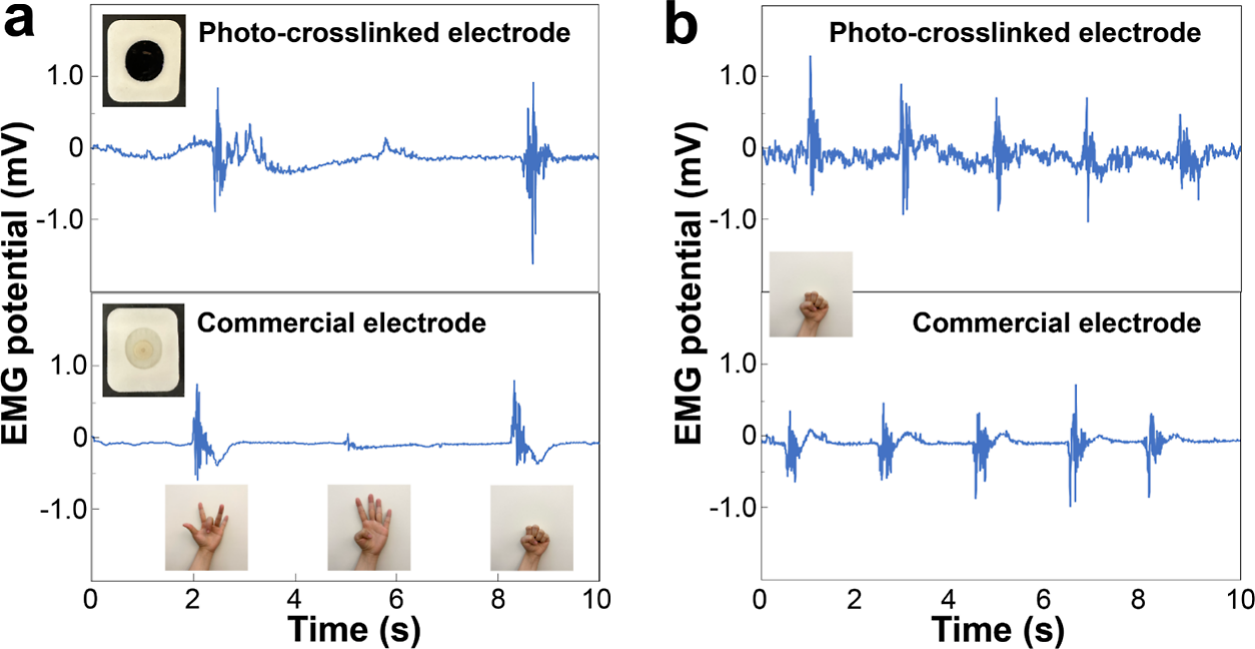
Comparison of the sEMG signals collected by a photo-cross-linked electrode (top) and a commercial 3 M electrode (bottom) adhered to the forearm (a) during different finger motions and (b) during repeated fist closing motions. The insets show photographs of the two electrodes and different finger/hand motions.

The photo-cross-linked hydrogels have good conductivities and elastic moduli similar to that of skin, which should enable conformal contact between the electrodes and skin.^15,16^ Thus, we demonstrated the application of the conductive hydrogels as wearable electrodes on the arm for surface electromyography (sEMG) to measure finger movements. We compared the photo-cross-linked hydrogels, SS:CoumAc:EDOT 100:20:13 (same formulation as above for the photopatterning), to a commercial 3 M Ag/AgCl hydrogel sEMG electrode (Figure 9). For ease of fabrication and wiring, the PEDOT-based conductive hydrogel was photo-crosslinked for 120 min directly into the electrode reservoir emptied from the commercial product. The electrodes adhered firmly onto the skin of the forearm to ensure good contact (Figure S13). The sEMG signals obtained with the photo-cross-linked hydrogel electrode closely resembled those recorded using commercial gel electrodes. The SNR (signal-to-noise ratio) of the sEMG signals collected by the photo-cross-linked electrodes was 10.7, compared to that of the commercial electrode, which was 11.1. The photo-cross-linked electrode, however, showed a slightly higher amplitude. The photo-cross-linked electrode showed good sensitivity by detecting changes in the sEMG signal when different finger movements (Figure 9a insets) and repeated fist closing motions (Figure 9b) were made, highlighting its potential as a wearable electrode.

## Conclusions

We have reported the first example of a one-pot, photocontrolled formation of conductive hydrogels, addressing the challenge of photo-cross-linking in the presence of a light-absorbing conducting polymer. This process was enabled by the synthesis of a photo-cross-linkable PSS-derived polymer, which served as a matrix for the polymerization of PEDOT. Compared with existing strategies to photo-crosslink conductive hydrogels, this approach did not involve the addition of an insulating scaffold around the conductive polymers but instead uses P(SS-*co*-CoumAc) as both the photo-cross-linkable scaffold and the counterion for doped PEDOT^+^. Moreover, it did not require degassing to remove oxygen since radical polymerizations are not involved in the process. Conductive hydrogels with good electronic and mechanical properties were produced, which were demonstrated for electrodes in the monitoring of sEMG signals. A proof-of-concept that the conductive hydrogels can be photopatterned was demonstrated to achieve microsized structures with high resolution. This method for forming conductive hydrogels in one step using light is expected to enable high-precision stereolithography and would provide soft conductive interfaces for wearable and implantable electrodes and tissue engineering scaffolds.

## Supporting Information Available

Materials purchased, instrumentations and methods, NMR spectra and SEC traces of P(SS-*co*-CoumAc) copolymers, UV–vis spectra, pictures of P(SS-*co*-CoumAc) solution and hydrogel, control experiments, XPS spectra, swelling and deswelling measurements, and pictures of electrodes placed on the human skin (PDF)
