# Supplementary material for: One Pot Photomediated Formation of Electrically Conductive Hydrogels
Source: ACS Polym Au. 2023 Dec 8;4(1):34–44. doi: 10.1021/acspolymersau.3c00031 (PMC10870748; doi:10.1021/acspolymersau.3c00031)
Supplement: Supplementary file 1 — lg3c00031_si_001.pdf [file lg3c00031_si_001.pdf]

Supporting Information

One Pot Photomediated Formation of Electrically Conductive Hydrogels

Dan My Nguyen,[1] Chun-Yuan Lo,[1] Tianzheng Guo,[2] Taewook Choi,[1] Shalini Sundar,[3] Zachary Swain,[2] Yuhang Wu,[2] Charles Dhong,[2,3] Laure V. Kayser*[1,2]

[1]Department of Chemistry and Biochemistry, University of Delaware, Newark, Delaware, 19716, USA
[2]Department of Materials Science and Engineering, University of Delaware, Newark, Delaware, 19716, USA
[3]Department of Biomedical Engineering, University of Delaware, Newark, Delaware, 19716, USA

*Author to whom correspondence should be addressed: lkayser@udel.edu

Materials

4-Methylumbelliferone, 2-bromoethanol, triethylamine, sodium 4-styrenesulfonate (NaSS), 4,4′-azobis(4-cyanovaleric acid) (ACVA), 3,4-ethylenedioxythiophene (EDOT), ammonium persulfate ($(NH_4)_2S_2O_8$), acidic resin (Dowex Marathon C hydrogen form) were purchased from Sigma-Aldrich and used without further purification. Potassium carbonate (K_2CO_3), anhydrous N,N-dimethylformamide (DMF), chloroform ($CHCl_3$), dioxane, dichloromethane (CH_2Cl_2), sodium sulfate (Na_2SO_4) were purchased from Fisher Scientific. Acryloyl chloride was purchased from Sigma-Aldrich and passed through a column of basic alumina to remove inhibitors and acidic impurities prior to the reaction. 3M™ Ag/AgCl Red Dot Monitoring Electrodes were purchased from Amazon.

General

Nuclear magnetic resonance (NMR). ^{1}H NMR spectrum were recorded on a Bruker 600 MHz spectrometer at room temperature.

Size exclusion chromatography (SEC). Number-average (M_n) and weight-average (M_w) molecular weights and dispersity ($Đ$) were determined by SEC using a Tosoh HLC-8420 GPC EcoSEC LC system equipped with a PSS GRAM column (10 µm, 8 ×300 mm) running in 10 vol% deionized water and 90% dimethylformamide (DMF) with 0.1 wt% lithium bromide (LiBr) at 25 °C with a flow rate of 0.8 mL min^{-1}, and calibrated against narrow dispersity polystyrene sulfonate standards (purchased from Polymer Standards Service).

UV-Vis Spectroscopy. UV-Vis measurements were taken using a Shimadzu UV-3600 Spectrometer at 25 °C. Absorbance measurements were conducted with 2 mL of sample in quartz cuvettes.

Photorheometer. The gelation process of the hydrogel precursor under UV light (365 nm) was evaluated by an oscillatory rheometer (AR-G2, TA Instruments) assembled with an OmniCure Series 2000 light source (25 mW cm^{-2}), a sample chamber, and an 8-mm diameter steel parallel-plate geometry. The testing was conducted at room temperature with a strain of 1% at 1Hz, which was found to be within the gel linear viscoelastic region, and an axial force control of 0 ± 0.5 N.

Electrochemical impedance spectroscopy (EIS). EIS measurements were performed on a Metrohm Autolab PGSTAT128N in a Faraday cage. Impedance magnitude and phase were scanned over a frequency range of 0.1 to $1x10^5$ Hz at 0 V bias with 10 mV amplitude.
Resistance measurements. The resistance was measured between two parallel stainless steel electrodes was measured using a Keithley 2400 source meter. The conductivity σ was calculated by using equation (1) where L is length, t is thickness, and w is width of hydrogel.

$$\sigma = \frac{L}{R \times t \times w} \qquad (1)$$

Tensile tests. The mechanical tests (stress-strain curves) on the conductive hydrogels (4 mm × 25 mm × 2 mm) were performed on a tensile tester (MTS, Tytron 250) at a speed of 9 mm min^{-1} at room temperature. Toughness was calculated from the integration of the stress-strain curves.

X-ray photoelectron spectroscopy (XPS). The elemental composition of the hydrogels was analyzed by X-ray photoelectron spectroscopy on a Thermo Scientific K-Alpha XPS system. The hydrogels were lyophilized and grinded into powder before taking XPS.

Scanning electron microscopy (SEM). Cross-sectional images were obtained using an Auriga 60 SEM. The hydrogels were frozen in liquid nitrogen, then cut in half with a razor blade, and finally lyophilized. Then the surface of the samples was sputter-coated with Palladium for 60 seconds before recording the SEM images.

Optical microscopy. Optical microscopy images of the photoprinted hydrogels were taken on a Zeiss Axiovert200M microscope.

Swelling and deswelling of the photo-cross-linked conductive hydrogels

In order to measure the swelling rate of the conductive hydrogel with SS:CoumAc:EDOT ratio of 100:20:13, its mass was measured at specific time intervals when submerged in 1X PBS solution at 25 °C. For the deswelling rate of the similar conductive hydrogel, its mass was measured at specific time intervals when left at 25 °C under ambient conditions. The weight percentage can be calculated from equation 2:

$$wt\% = \frac{m_d}{m_o} \times 100\% \quad (2)$$

where m_d is the mass measured, and m_o is the original mass.

Electrode fabrication and surface electromyography (sEMG) measurements
Photo-cross-linked electrode fabrication. To fabricate photo-cross-linked electrodes, we carved out the gels in the commercial 3M™ Ag/AgCl Red Dot Monitoring electrodes. Then, we placed the precursor solution with a SS:CoumAc:EDOT ratio of 100:20:13 into the electrode mold and photo-irradiated it for 120 min. The photo-cross-linked gels were then washed multiple times with deionized water and PBS before conducting the sEMG measurements.

sEMG measurements. The EMG measurements were performed using the methods described by Blau *et. al.*[1] The electrodes were adhered firmly onto the skin of the forearm to ensure good contact (**Figure S5**). The electrodes were connected to the ECG channel of a MAX30001- EVSYS evaluation board (Maxim Integrated Products, *Inc*.). The reference electrode (3M^{TM} Ag/AgCl Red Dot Monitoring Electrode) was placed on the elbow of the same arm and connected to the Body Bias pin of the board. The ECG channel was set with a gain of 20 V/V and a sampling rate of 512 readings s^{-1}. Post-ADC digital filters were used during the recordings. The low pass filter cutoff was 40 Hz, and the high pass filter cutoff was 0.5 Hz. A Butterworth notch filter with a width of 59 to 61 Hz was applied post-measurement to eliminate 60 Hz power-line noise. The signal-to-noise ratio (SNR) was calculated from the root mean square (RMS) of the filtered data and the ratio between peaks and their base line.

Laser writer

The patterned polymer was made by laser writing using a commercial maskless photolithography tool (Smart Print UV, microlight3D). The pattern was designed in the mask design software Klayout. Then the pattern file was imported into Phaos, which is a dedicated software to configure the maskless photolithography tool. The designed pattern was split into 18 stitches and the exposure time was set to 45 seconds for each stitch. The precursor ink solution with a SS:CoumAc:EDOT ratio of 100:20:13 was spread onto a microscope glass slide (Fisher Scientific) and the glass slide was placed onto the exposure stage of the photolithography tool. The wavelength of the UV light was 385 nm and the light power density was 2072 mW cm^{-2}.

Supplementary figures

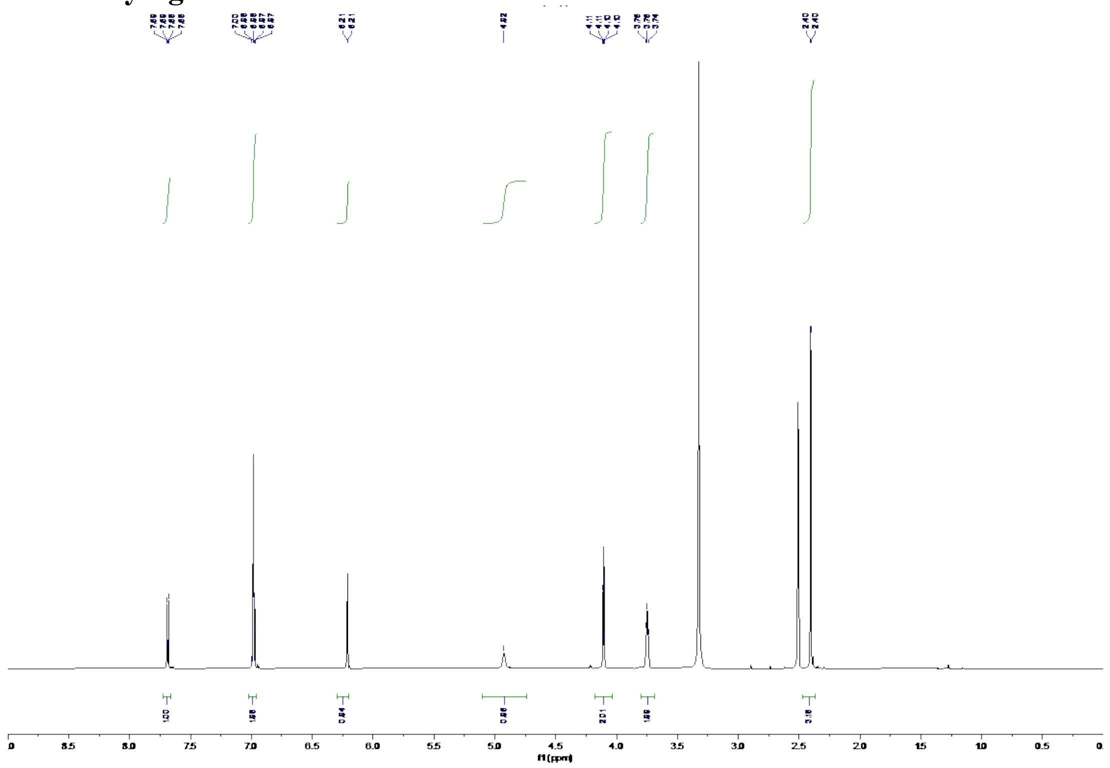

Figure S1. ^{1}H NMR spectrum of 7-(2-hydroxyethoxy)-4-methylcoumarin (CoumOH) in DMSO-d_6

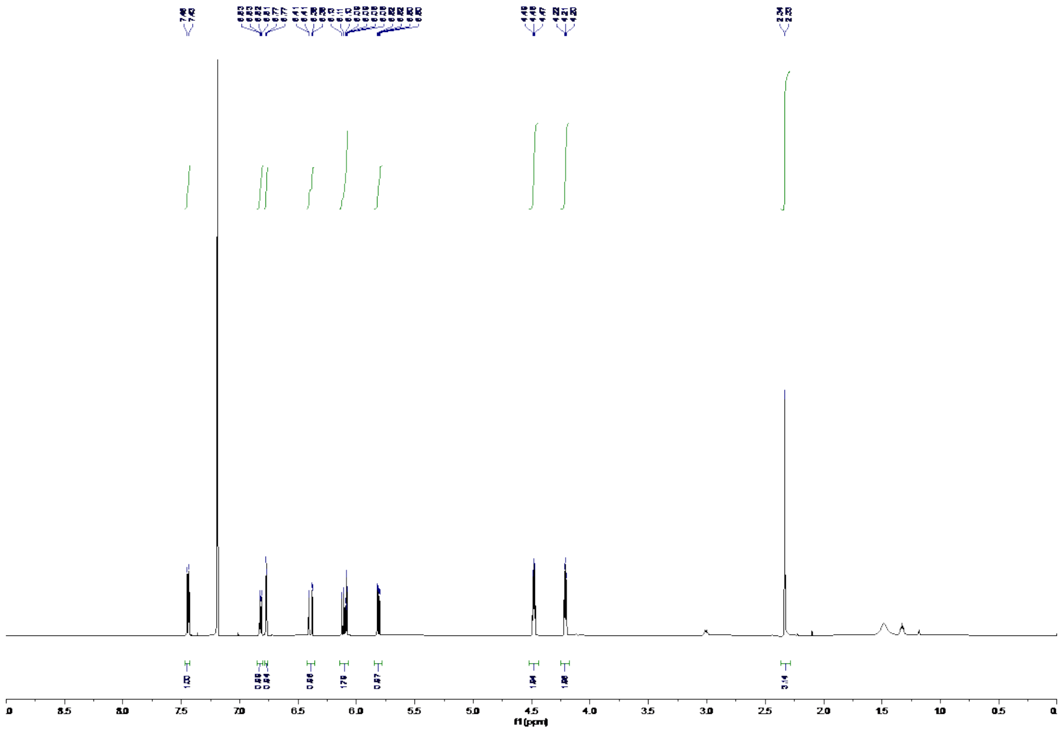

Figure S2. ^{1}H NMR spectrum of 7-(2-acryloyloxyethoxy)-4-methylcoumarin (CoumAc**)** in $CDCl_3$

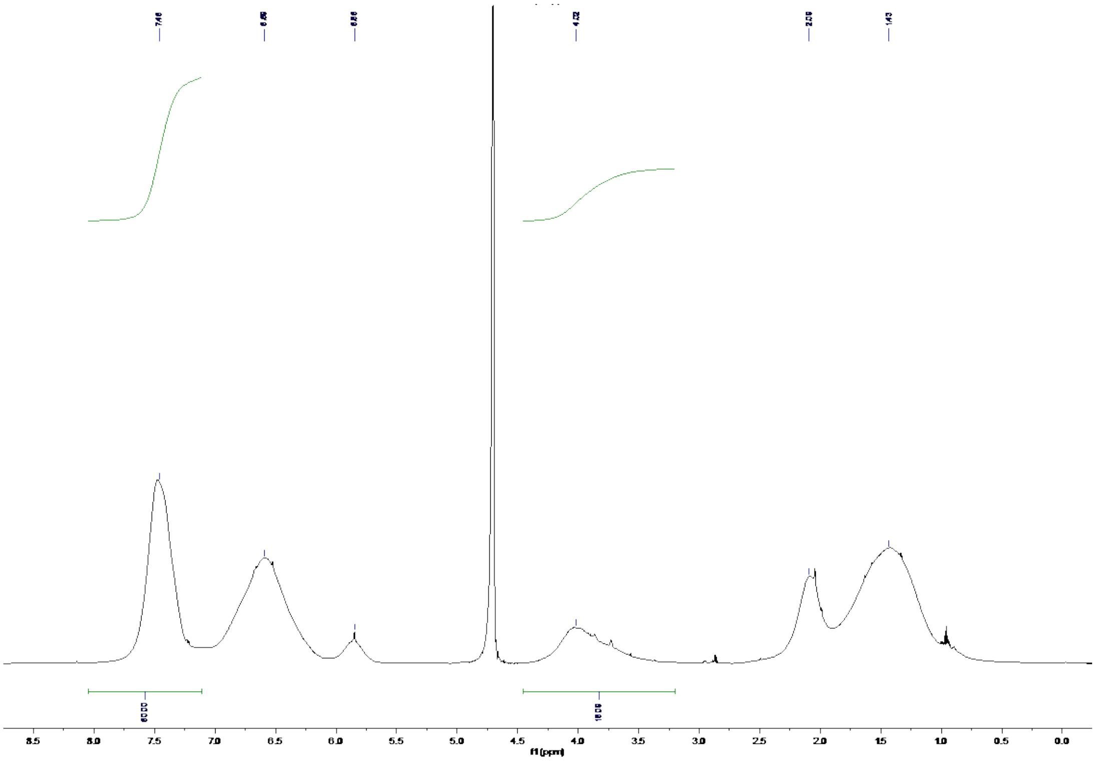

Figure S3. ^{1}H NMR spectrum of P(NaSS-*co*-CoumAc) with PSS to PCoumAc ratio of 100:20 in D_2O.

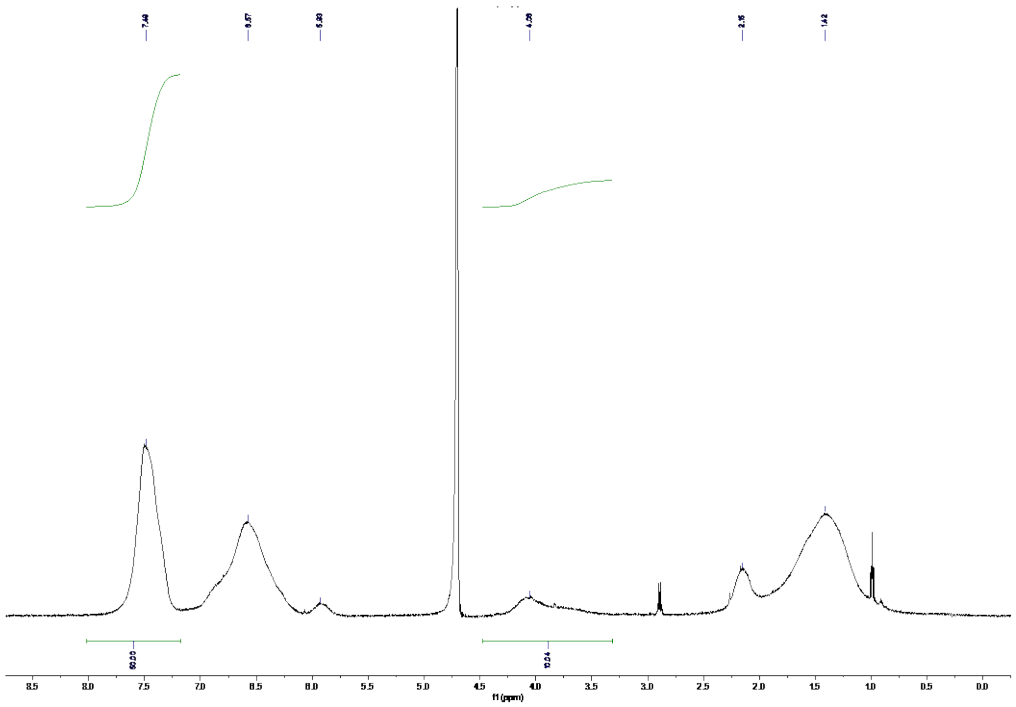

Figure S4. ^{1}H NMR spectrum of P(NaSS-*co*-CoumAc) with PSS to PCoumAc ratio of 100:10 in D_2O.

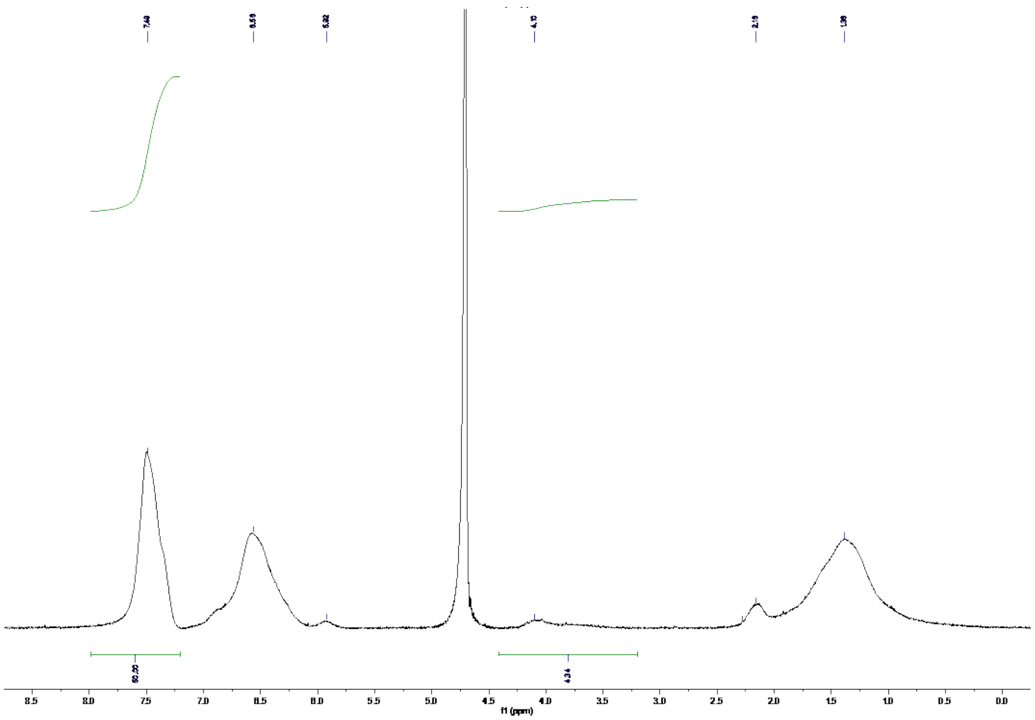

Figure S5. ^{1}H NMR spectrum of P(NaSS-*co*-CoumAc) with PSS to PCoumAc ratio of 100:5 in D_2O.

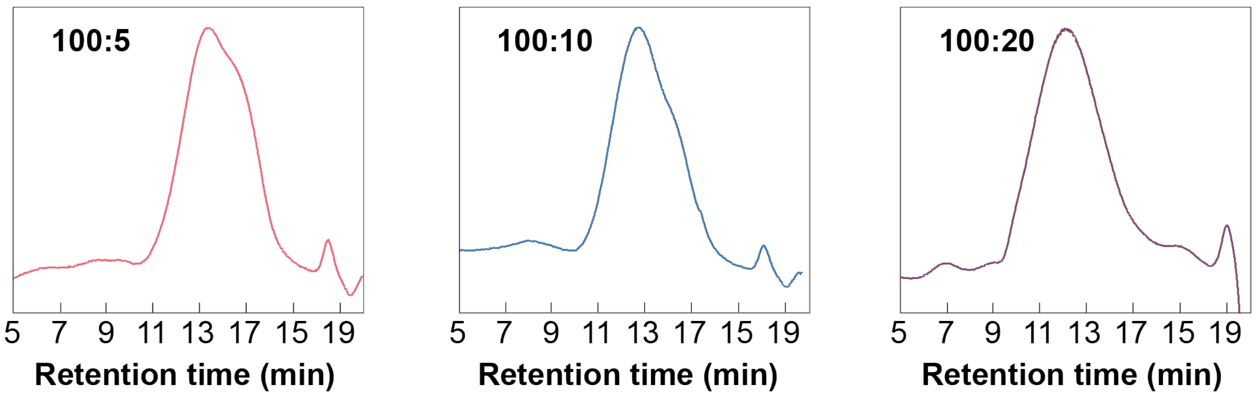

Figure S6. SEC traces of P(NaSS-*co*-CoumAc) with a SS:CoumAc cross-linker ratio of 100:5, 100:10, and 100:20.

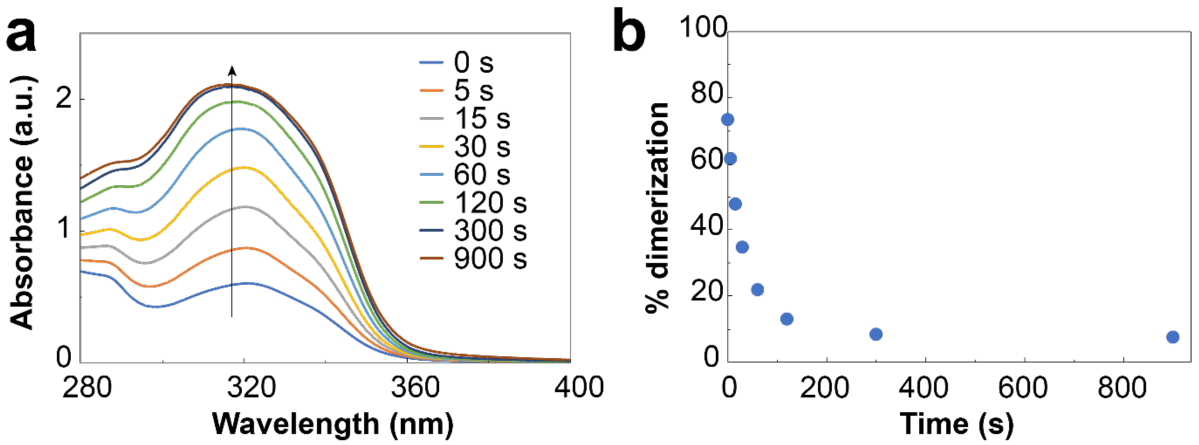

Figure S7. UV-Vis study of the photo-cleavage of P(SS-*co*-CoumAc). (a) UV-Vis spectra over 900 s (15 min) of irradiation at 254 nm of a solution of P(SS-*co*-CoumAc) copolymer (0.05 wt%). (b) Percent coumarin de-dimerization as a function of irradiation time at 254 nm.

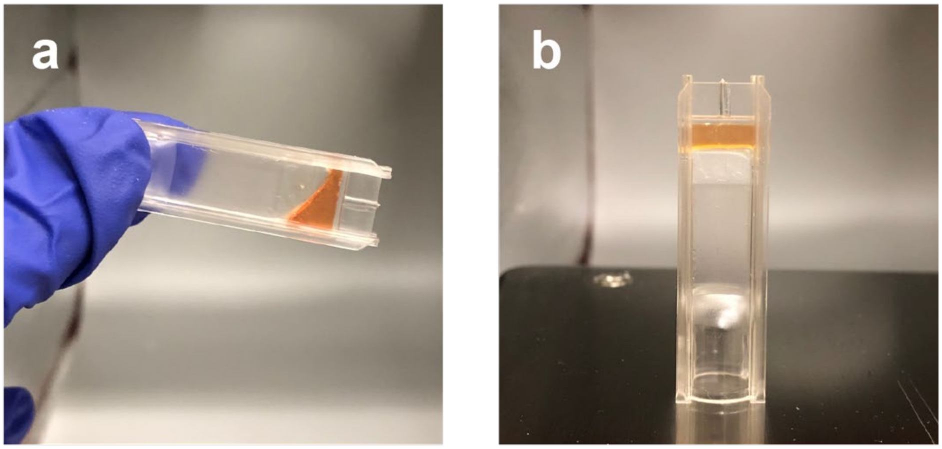

Figure S8. Picture of **(a)** the precursor solution of P(SS-*co*-CoumAc) with a 100:10 SS:CoumAc ratio and **(b)** the resulting hydrogel after light irradiation for 120 minutes.

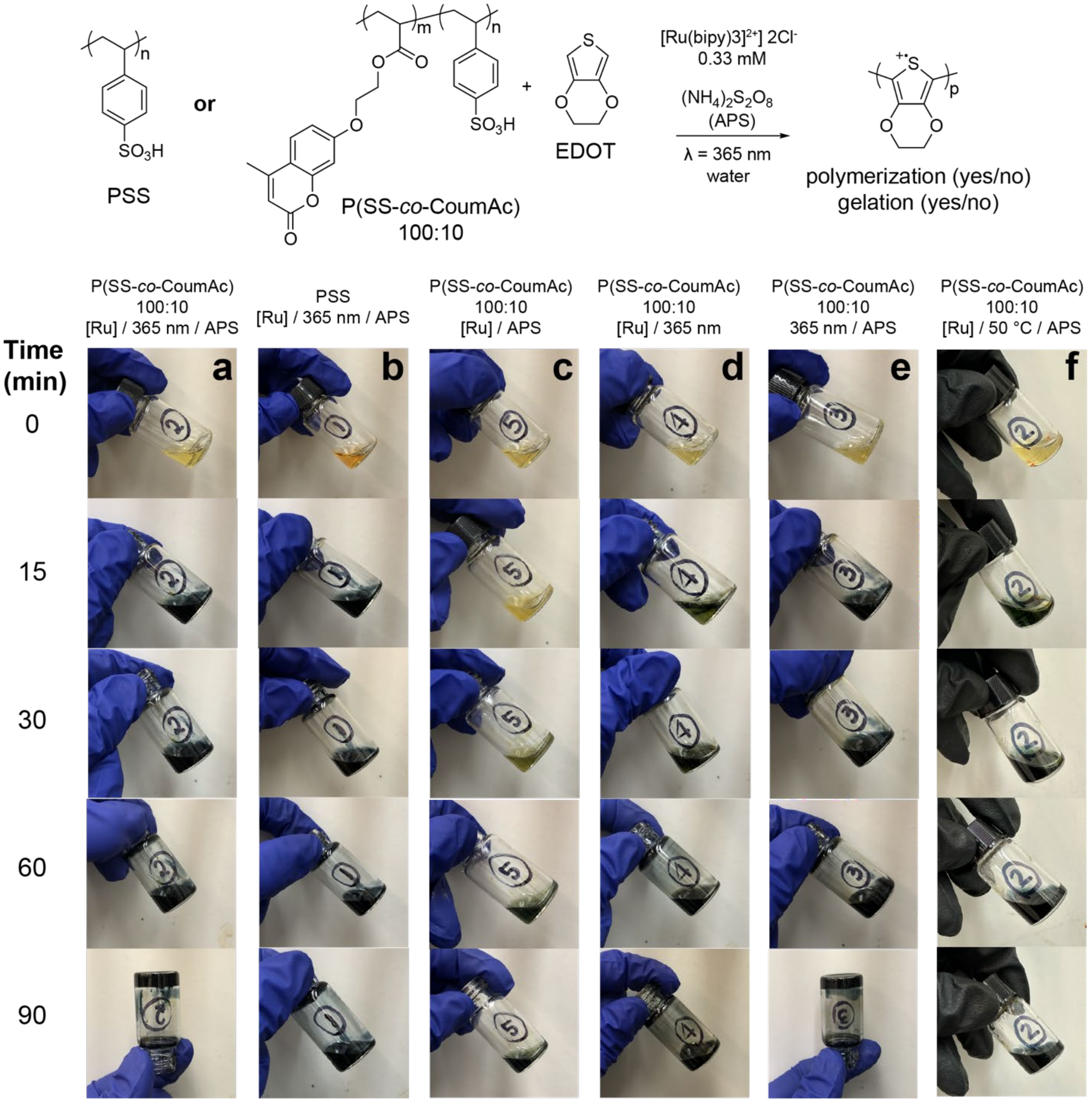

Figure S9. Control experiments. **(a)** With P(SS-*co*-CoumAc), ammonium persulfate (APS), ruthenium catalyst, and light irradiation. **(b)** Same conditions as (a) but with PSS. **(c)** Same conditions as (a) but without light. **(d)** Same conditions as (a) but without APS. **(e)** Same conditions as (a) but without ruthenium catalyst. **(f)** Same conditions as (a) but without light and with heating at 50 °C.

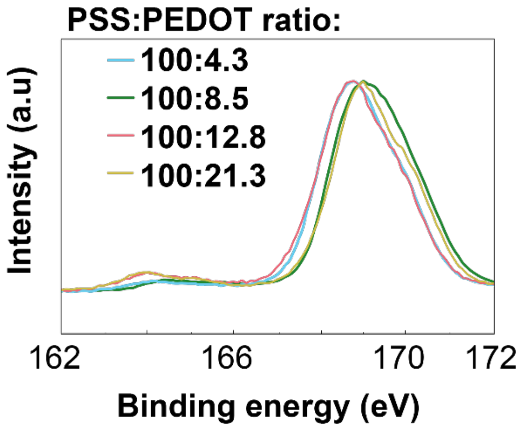

Figure S10. S(2p) X-ray photoelectron spectroscopy (XPS) spectra of all conductive hydrogels.

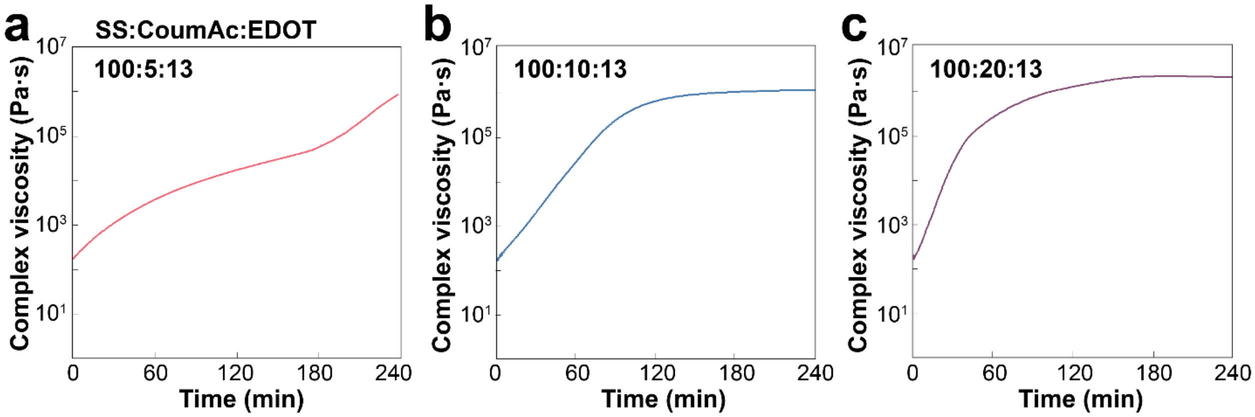

Figure S11. Change in viscosity as function of irradiation time.

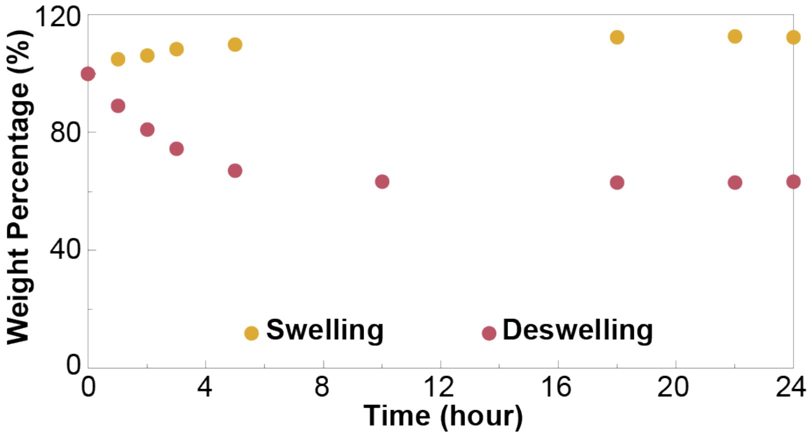

Figure S12. Swelling (in 1X PBS) and deswelling (under ambient conditions) measurements of the conductive hydrogel with SS:CoumAc:EDOT ratio of 100:20:13 over 24 hours.

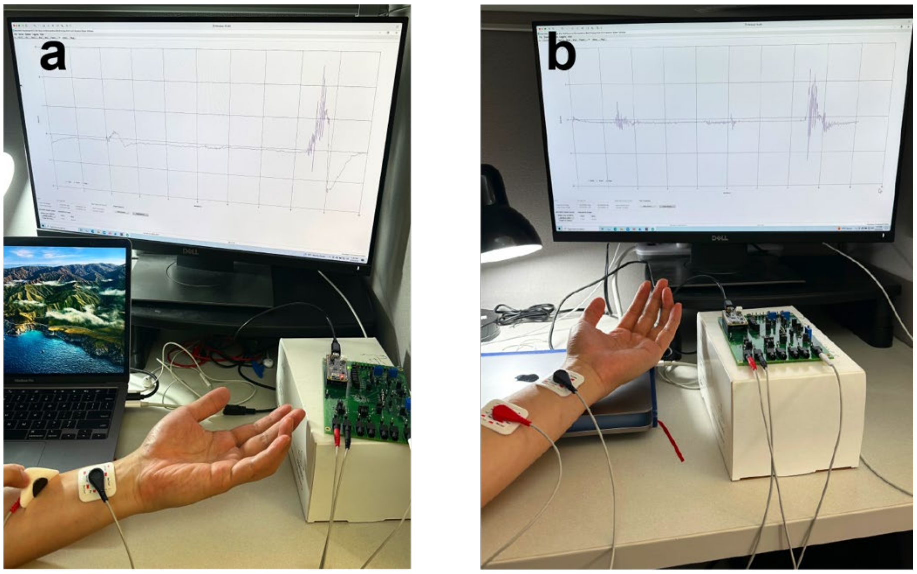

Figure S13. Pictures of electrodes adhered onto the skin of the forearm **(a)** Photo-cross-linked electrodes and **(b)** Commercial 3M^{TM} electrode

References

1. Blau, R.; Chen, A. X.; Polat, B.; Becerra, L. L.; Runser, R.; Zamanimeymian, B.; Choudhary, K.; Lipomi, D. J., Intrinsically Stretchable Block Copolymer Based on PEDOT:PSS for Improved Performance in Bioelectronic Applications. *ACS Appl. Mater. Interfaces* **2022,** *14* (4), 4823-4835.
